# Endodontic Retreatment of a Maxillary First Molar With Orthograde Retrieval of a Separated File by a Combination of Ultrasonic and File Braiding Techniques: A Case Report

**DOI:** 10.7759/cureus.50140

**Published:** 2023-12-07

**Authors:** Mohit Zarekar, Apurva Satpute, Mohini Zarekar

**Affiliations:** 1 Paedodontics & Preventive Dentistry, Private Practice, Ahmednagar, IND; 2 Conservative Dentistry and Endodontics, Government Dental College & Hospital, Aurangabad, Aurangabad, IND; 3 Institute of Tropical Medicine, Charité – Universitätsmedizin Berlin, Berlin, DEU

**Keywords:** maxillary first molar, braiding technique, ultrasonic, file retrieval, retreatment

## Abstract

The persistence of apical periodontitis after endodontic therapy can be attributed to biological causes or when the treatment approaches have proven insufficient in completely eradicating the intra-radicular infection. This case report describes the endodontic retreatment of a maxillary molar in which file separation occurred during the cleaning and shaping procedure. The separated file was successfully retrieved utilizing a combination of ultrasonic and braiding techniques. The field of ultrasonics has undergone a thorough investigation and has been demonstrated to be a conservative technique with minimal radicular dentin trephination. The braiding technique is also a conservative method that involves the use of several braided H-files to apply a gripping force, facilitating the extraction of foreign bodies. The utilization of this methodology has been documented in the endodontic academic literature.

## Introduction

Retreating a tooth that has undergone prior endodontic treatment is a frequently encountered situation within the field of dentistry [1]. However, there is limited information regarding endodontic retreatment in the existing literature. The lack of recognition of the distinctions between endodontic retreatment and initial endodontic treatment may be a contributing factor. Several retrospective investigations have provided data demonstrating a success rate ranging from 53% to 96%. The higher numerical value implies a high success rate for endodontic treatments, whereas the lower numerical value suggests a relatively lower success rate, with approximately half of the cases resulting in failure [1,2]. The prognosis often worsens as the number of retreatment procedures increases. Various meta-analyses have found several significant factors for the success of retreatment. These include the preoperative periapical status, size of the lesion, apical extent of root filing, and quality of coronal restoration [3]. A systematic investigation of the impact of study features and clinical factors on retreatment outcomes has not yet been conducted. This information would be beneficial for informing clinical decision-making regarding potential retreatment alternatives [4]. The primary aim of endodontic retreatment is to effectively decontaminate the root canals by removing irritants, particularly microbes that have either persisted or invaded the canals subsequent to prior treatment. Retreatments are recommended whenever feasible, as they entail the repetition of root canal therapy stages with a biological justification [5]. Understanding the etiology of failure is crucial in the management of teeth affected by post-treatment disease, as it enables the identification and appropriate resolution of the underlying cause [6]. Moreover, the existence of fragmented endodontic instruments within the root canal system adversely impacts the prognosis of endodontic retreatment [7]. Based on the majority of prior research, the utilization of nickel-titanium rotary files is associated with a higher susceptibility to separations compared to manual stainless-steel files. This is primarily attributed to the hindrance these rotary files pose in terms of effectively cleaning and shaping the apical root canal [7,8]. The management of separated endodontic instruments encompasses several approaches based on their location and relationship to the curvature. These approaches include non-surgical retrieval, bypassing, follow-up, and surgical treatment. There is a correlation between elevated rates of treatment failure and the presence of unremoved instruments obstructed within canals that already have underlying periapical lesions [9]. The retrieval of separated instruments is sometimes challenging and perhaps unattainable, as evidenced by a reported success rate ranging from 55% to 79% [10]. The existing body of literature consists of a solitary case report that examines the management of a previously treated maxillary first molar. This case involved the occurrence of file separation, which was subsequently retrieved through the utilization of ultrasonic and the file braiding approach which involves the insertion of several H-files and their subsequent twisting around the foreign body (separated file). In this technique, H-files are the ideal choice due to their flute design, which is well-suited for engaging the separated file. The braided multiple H-files would apply a strong grasping force that facilitates the extraction of the separated file.

## Case presentation

A 31-year-old male patient presented with symptoms of pain and swelling in the upper left maxilla region. The intensity of the pain was throbbing at night, causing disruptions in his sleep patterns, and proving resistant to medications. The patient presented with a recurring, minor swelling occurring once or twice a year within the same localized region over two years. The endodontic treatment had been performed on this tooth four years ago.

**Figure 1:**
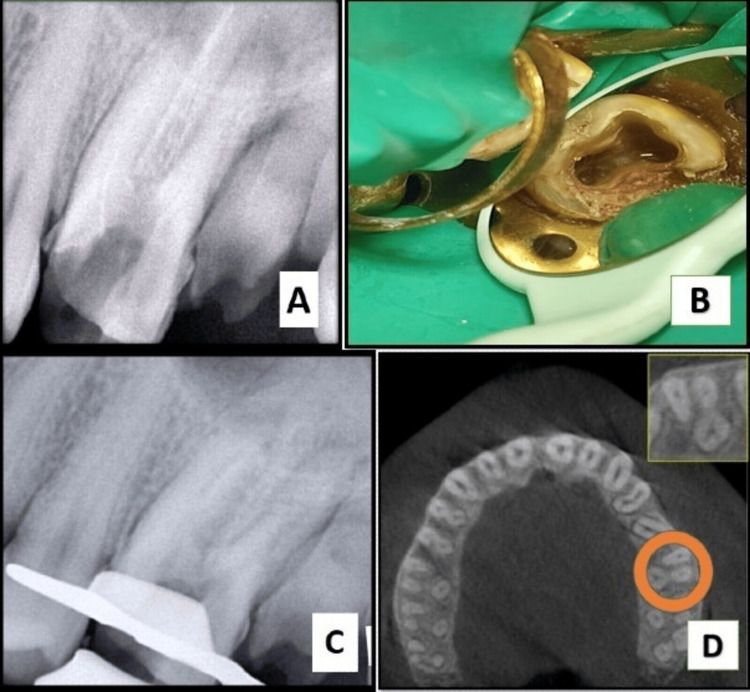
(A) Preoperative radiograph showing inadequate obturation and loss of coronal restoration. (B) Redefining the access cavity. (C) Gutta-percha removal. (D) Cone-beam computed tomography image showing a single canal in the mesiobuccal root.

**Figure 2:**
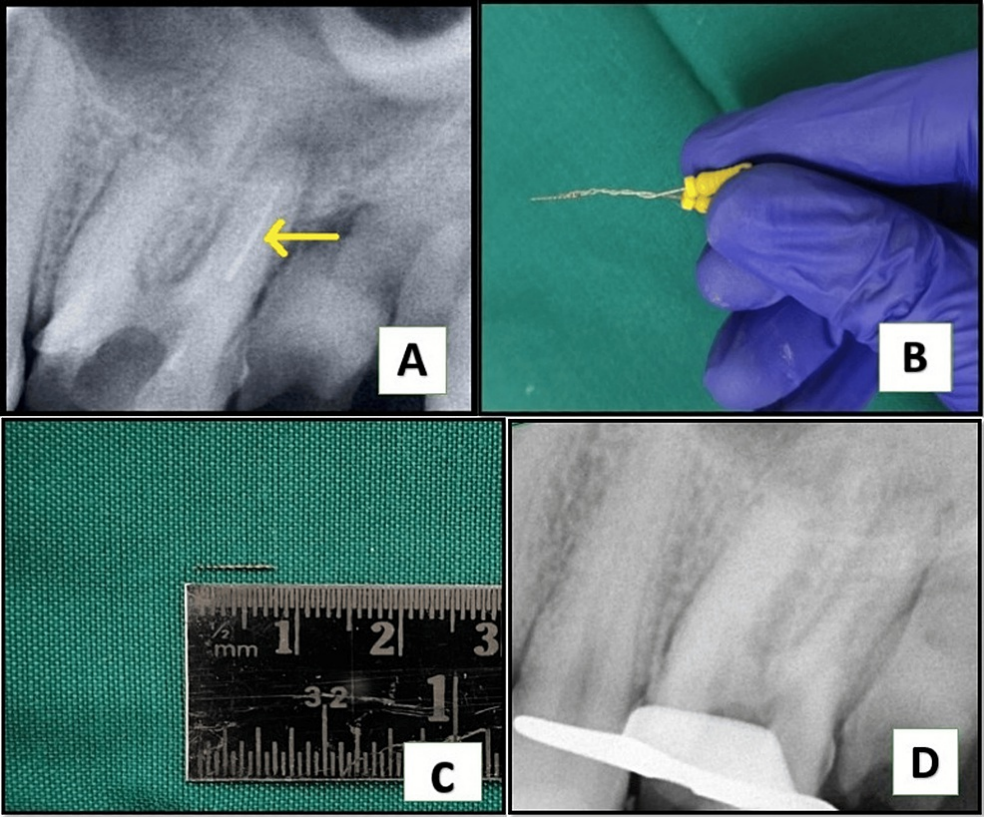
(A) Radiograph showing the separated instrument in the distobuccal canal. (B) Retrieval of the instrument seen clinically with the braiding technique. (C) Measurement of the separated file. (D) Post-retrieval radiograph.

**Figure 3:**
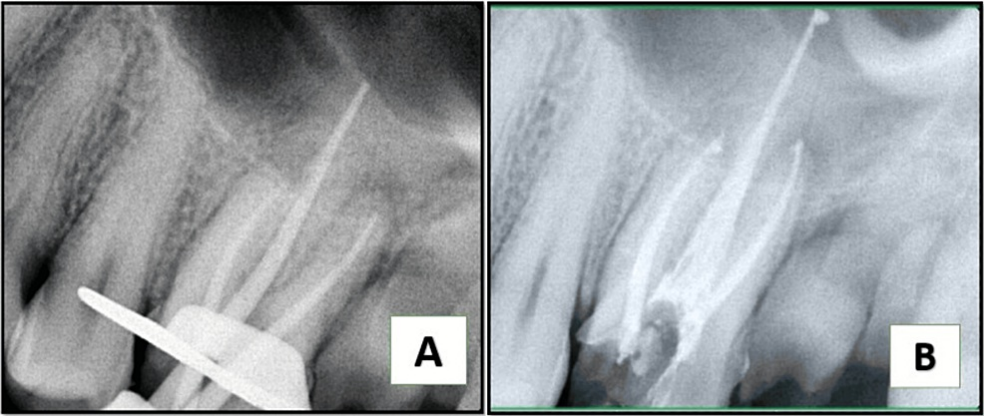
(A) Mastercone radiograph. (B) Immediate post-obturation radiograph.

**Figure 4:**
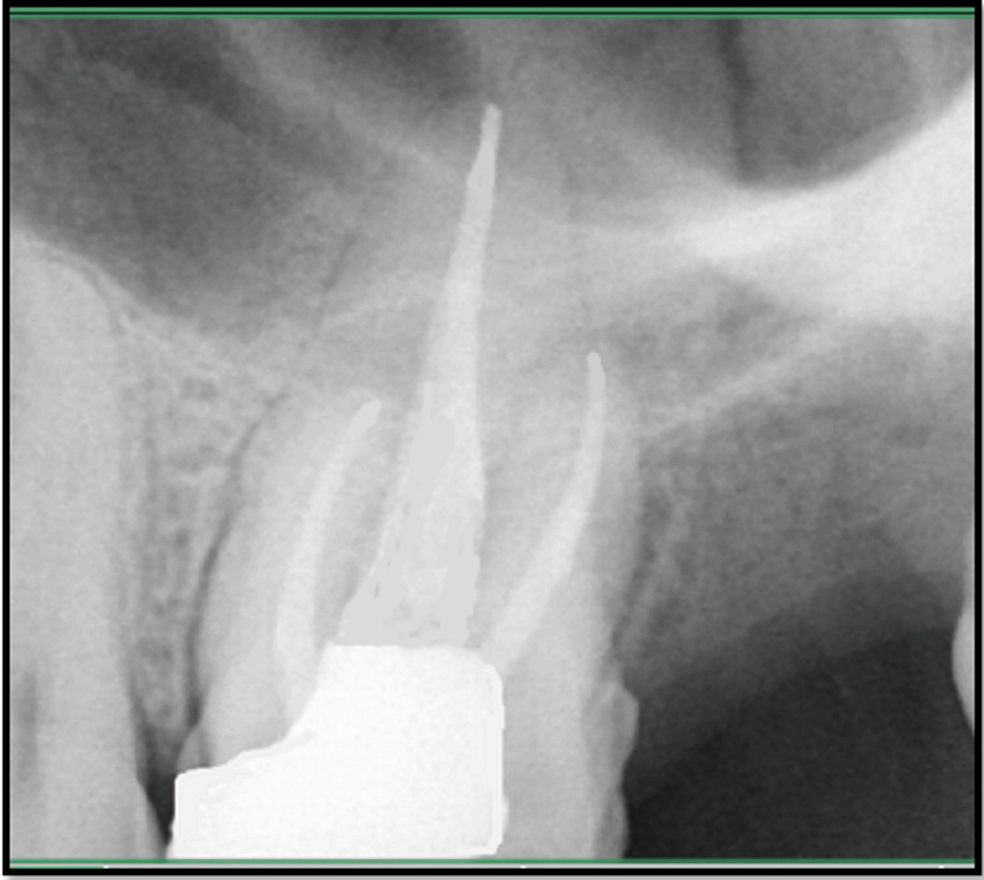
One-year follow-up radiograph showing sufficient healing.

On intraoral examination, a fluctuant swelling was observed on the buccal mucosa in close proximity to tooth #26, which had extensive dental caries. Notably, the affected area exhibited heightened sensitivity upon palpation. The coronal restoration was displaced. The measurements of periodontal probing depths were within the normal range. The radiographic analysis indicated that tooth #26 had inadequate obturation in the mesiobuccal, distal, and palatal canals, accompanied by the presence of periapical radiolucency (Figure 1A). Tooth #26 was diagnosed with acute suppurative apical periodontitis. After a thorough evaluation of the patient’s clinical signs and radiographic findings, it was determined that a non-surgical endodontic retreatment was the most appropriate course of action. The patient was provided with a detailed explanation of the treatment protocol and subsequently agreed to proceed. Before initiating a non-surgical endodontic retreatment, thorough consideration was given to alternative multidisciplinary treatment methods, which were subsequently deemed ineffective. Upon obtaining informed consent from the patient, it was determined to proceed with the retreatment operation. After administration of the suitable anesthesia and isolation with a rubber dam, access was regained (Figure 1B). H-files (Dentsply, Maileffer, USA) were used to remove the loosely packed root filling, together with the use of GP solvent (GP Cleanse, Deor) to facilitate the softening of the gutta-percha before the utilization of H-files (Figure 1C). A purulent discharge was evacuated through the access cavity and facilitated by the use of a sterile saline flush administered via a side vent needle (EndoTop, Cerkamed, Stalowa Wola, Poland). Working length was established by employing an apex locator (Root ZX, J. Morita Inc., USA). The canals were meticulously prepared using manual hand k-files (Dentsply, Maileffer, USA) and RaCe NiTi rotary files, following a crown-down technique. Various clinical techniques and methodologies have been employed to improve the visualization of the MB2 canal in the mesiobuccal root. However, these endeavors have not yielded successful results. Consequently, we recommended that the patient undergo a cone-beam computed tomography scan to obtain an accurate anatomical three-dimensional image (Figure 1D), facilitating the identification of the MB2 canal. Subsequently, it was determined that only one canal existed in the mesiobuccal root, so we proceeded with the preparation of all three canals using the crown-down approach. During cleaning and shaping, approximately 8 mm of a size #0.04/20 RaCe NiTi file separated within the distobuccal canal. A radiographic image was obtained to verify the extent of the instrument’s separation (Figure 2A). The instrument was observed to be within the middle third of the distobuccal canal. The proposed procedure was the retrieval of the instrument, which was accomplished by preparing a staging platform at the most coronal aspect of the fragment using modified Gates Glidden burs (no. 2-4). The ProUltra ultrasonic tip No. 4 (Dentsply Tulsa Dental in Tulsa, OK) was utilized at reduced power levels at no. 6 setting to effectively remove dentin and send vibrations to dislodge the separated fragment. The experimental protocol was conducted using magnifying loupes with a magnification factor of 2.5 (Heine, Germany). Intermittent irrigation with normal saline was performed to effectively remove debris from the canal and provide a cooling effect. After approximately 40 minutes of applying ultrasonic tips and sufficient irrigation, the instrument loosened but did not completely disengage from the canal despite the use of copious amounts of irrigation. Consequently, we opted to utilize a different approach known as the file braiding technique which employs multiple H-files braided around separated files that would apply a strong grasping force, enabling the extraction of a separated file. This method is successful when the fragment is located deep within the canal and is not visible. The operator relies on tactile sensation or when the fragment is loose but cannot be retrieved using other methods such as microtube extraction devices, ultrasonics, or pliers/forceps. Two no. 20 H-files were introduced, one from the buccal side and the other from the lingual side. The files were then twisted together clockwise to secure the file section inside the canal. After rotating in a clockwise direction, they were extracted from the canal. The separated file segment emerged from the canal in conjunction with the H-files (Figure 2B) which was measured to be around 8 mm (Figure 2C). The radiographic image was obtained after the successful retrieval of the separated file (Figure 2D). After the application of calcium hydroxide (CH) paste as an intracanal dressing, the patient was scheduled for a follow-up appointment two weeks later. During the subsequent examination, there were no apparent indications of discomfort, tenderness, or edema. The root canal was treated using a combination of 2.5% sodium hypochlorite ultrasound-activated irrigation and negative apical pressure facilitated by the EndoVac device to effectively remove the calcium hydroxide paste. The canal was dried using a sterile paper point and subsequently sealed using appropriate gutta-percha master cones (Figure 3A) and DIA-ROOT BIO (DiaDent Group International, South Korea) bioceramic sealer (Figure 3B). After the obturation procedure, the tooth was subsequently rebuilt using a resin composite restoration (MultiCore Flow, Ivoclar Vivadent). A follow-up radiograph taken after one year showed sufficient healing (Figure 4).

## Discussion

Although selective root canal retreatment has been used clinically for a considerable period, its documentation in the scientific literature as a viable technique for managing post-treatment pathology in teeth has only been documented recently [6]. Non-surgical endodontic treatment is generally recommended in cases where the initial root canal treatment proves ineffective. According to a previous study, preoperative periapical diagnosis is a highly influential factor in predicting the outcome of endodontic procedures. Moreover, the study revealed a correlation between the diagnosis of periradicular diseases and varying levels of risk. The unfavorable impact on endodontic outcomes is most significantly influenced by the existence and size of a preoperative periapical lesion [11]. Torabinejad et al. (2009) conducted a study to assess the efficacy of surgical and non-surgical retreatment approaches. The findings revealed that non-surgical retreatment exhibited a much higher success rate of 83% in contrast to the surgical method, which yielded a success rate of 71.8%. The data indicates that non-surgical endodontics exhibited an upward trend in weighted success over time, but endodontic surgery demonstrated a clear downward trajectory [12]. The failure of the initial treatment is frequently indicated by symptoms such as increased sensitivity to percussion, experiencing discomfort to thermal changes, pain on palpation or while mastication reported by the patient, and/or the persistent presence of a periapical lesion identified by the dentist. It is commonly acknowledged that the primary cause of unsuccessful cases is the existence of intra-radicular or extra-radicular infection [13]. The occurrence of file separation during endodontic therapy is a challenging event, with reported rates ranging from 2% to 6% in examined instances. The presence of a fragmented obstruction impedes the ability to perform a comprehensive root canal cleaning and shaping technique [14]. Various ways have been documented in the literature for the retrieval of split files, such as the Masserann kit, Endo Extractor, wire loop technique, braiding technique, and ultrasonics [15]. The orthograde technique involves three distinct phases: bypassing the instrument, removing the instrument, and preparing the canal and obturating it up to the level of the separated instrument [16]. However, in our specific scenario, it is important to consider the presence of a periapical infection. Therefore, it is required to bypass or retrieve the separated instrument. Simply bypassing or obturating to the level of the fractured instrument will not effectively achieve the goal of proper disinfection and cleansing. Consequently, we decided to attempt the retrieval of a separated instrument.

Ultrasonics are often regarded as the most conservative approach for elimination, making them the most extensively studied and utilized technique in both laboratory settings and living organisms. The technique frequently described involves the utilization of a modified Gates-Glidden drill to establish a staging platform. This platform allows for adequate space, enabling specialized ultrasonic tips to perform trephination around the coronal aspect of the fragment. Consequently, this process agitates, loosens, and unwinds the fractured instrument [17]. The retrieval of the instrument using either the Masserann kit or the Endosicherheits system necessitates the construction of an access canal with a minimum diameter of 1.2 mm. These operations carry a significant risk of root perforation as a substantial amount of dentin must be removed to properly position the extractor to retrieve the separated file. This is due to the use of a relatively large diameter tube compared to the size of the canal and subsequent reduction in radicular dentin [18]. This study employed ultrasonics as the initial method for accessing the separated instrument. This was achieved by creating a staging platform, followed by the retrieval of the instrument using a braiding technique that involves the insertion of numerous H-files, which are subsequently twisted around the foreign body. Throughout the procedure, careful consideration was given to preserving the integrity of the radicular dentin. In recent times, several studies have put forth the notion that the endodontic outcome of retrograde and orthograde root canal retreatment is comparable in retreatment procedures [19]. However, there is a pressing need to investigate the impact of several untested preoperative prognostic variables that are specifically associated with second root canal therapy. These variables include tooth location, size of periapical radiolucency, quality of the coronal restoration, magnification/illumination, disinfection of gutta-percha, time taken to place the definitive coronal restoration, and density of the root canal filling. Moreover, it is crucial to conduct randomized controlled studies that include comprehensive data recording to develop the most effective retreatment regimens [20].

Clinical decision-making algorithm in case of fractured instruments within root canal

The conventional method for addressing a separated instrument is to retrieve it. The global acceptance of utilizing an operating endomicroscope to aid in visualizing and shaping the canal up to the point of the fractured fragment and subsequently extracting it using ultrasonic tips and/or appropriate grasping tools is well-established. The position of a separated fragment can have an impact on the decision-making process.

If the instrument is fractured at the apical third of the root with a vital pulp, it is recommended to adjust the working length, prepare the canal up to the fragment, and employ sodium hypochlorite (NaOCl) agitation. Care should be taken not to extrude beyond the root canal into peri-radicular tissues as it causes chemical burns leading to localized or extensive tissue necrosis. A severe acute inflammatory reaction of tissue may develop. It is more desirable to perform obturation up to the fragment rather than attempting to retrieve it. In the case of non-vital (infected) pulp, it is not recommended to routinely attempt the removal of the fractured file fragment if substantial cleaning and shaping have been performed. It is advisable to obturate up to the fragment. Otherwise, it is highly suggested to bypass the fragment.

If the fragment is separated in the middle third with vital pulp. It is advisable to bypass the file. If bypassing is not feasible, one can obturate the canal up to the instrument. Follow-up is required, and in the event of post-treatment endodontic disease, periapical surgery should be employed. In the case of non-vital pulp, one should attempt to bypass and subsequently attempt to retrieve that file with minimal dentin removal. If it is not possible to bypass, the canal should be completely sealed up to the instrument and further treatment should involve considering apical surgery.

If an instrument becomes separated at the coronal third with vital or non-vital pulp, the broken instrument should be removed with minimal dentin removal. Various forms of gripping equipment such as Stieglitz forceps or plier-type instruments can be applied for this purpose.

## Conclusions

The effectiveness of endodontic therapy is contingent upon several key factors, including the quality of the coronal restoration, the supply of a durable coronal seal, thorough debridement and disinfection, and the three-dimensional obturation of a root canal. The implementation of sufficient coronal restorations is crucial in preventing leakage from the oral cavity into the canal system, which can lead to the disruption of the apical seal and subsequent treatment failure. Furthermore, the determination of the effectiveness of a method for instrument retrieval is a multifaceted task. Before initiating instrument removal, it is imperative to conduct a comprehensive assessment of the prevailing circumstances and carefully evaluate the potential hazards involved. Several factors need to be considered, including the morphology of the root canal, the condition of the tooth structure’s restorations, the availability of necessary equipment, and the prognosis of the case, to achieve a favorable result.
